# Clinical Application of Mindfulness-Oriented Meditation: A Preliminary Study in Children with ADHD

**DOI:** 10.3390/ijerph17186916

**Published:** 2020-09-22

**Authors:** Ornella Santonastaso, Vittoria Zaccari, Cristiano Crescentini, Franco Fabbro, Viviana Capurso, Stefano Vicari, Deny Menghini

**Affiliations:** 1 Child and Adolescent Neuropsychiatry Unit, Department of Neurological and Psychiatric Science, Bambino Gesù Children’s Hospital, 00165 Rome, Italy; ornella.santonastaso@libero.it (O.S.); zaccarivittoria89@hotmail.it (V.Z.); stefano.vicari@opbg.net (S.V.); 2 Department of Languages and Literatures, Communication, Education and Society, University of Udine, 33100 Udine, Italy; cristiano.crescentini@uniud.it (C.C.); franco.fabbro@uniud.it (F.F.); viviana.capurso@uniud.it (V.C.); 3 Department of Life Science and Public Health, Università Cattolica del Sacro Cuore, 00168 Rome, Italy

**Keywords:** mindfulness meditation, attention-deficit/hyperactivity disorder, neurodevelopmental disorders, neuropsychological measures

## Abstract

Mindfulness-oriented meditation (MOM) is a self-regulatory training used for attentional and behavioral problems. With its focus on attention, MOM is a promising form of training that is gaining empirical support as a complementary or alternative intervention for attention deficit/hyperactivity disorder (ADHD). In this study, we tested the preliminary efficacy of MOM training in children with ADHD, by comparing its efficacy with an active control condition (Emotion Education Program, EEP). Twenty-five children with ADHD aged 7–11 years participated in MOM training (*n* = 15) or EEP (*n* = 10) 3 times per week for 8 weeks. Neuropsychological and academic measures and behavioral, emotional, and mindfulness ratings were collected before and after the two programs. On average, MOM training had positive effects on neuropsychological measures, as evidenced by a significant mean improvement in all outcome measures after training. Moreover, positive effects on ADHD symptoms were found only in the MOM group. Although they are preliminary, our results documented that MOM training promotes changes in neuropsychological measures and in certain behavioral symptoms, suggesting it as a promising tool for ameliorating cognitive and clinical manifestations of ADHD.

## 1. Introduction

Attention deficit/hyperactivity disorder (ADHD) is one of the most common developmental disorders, with high persistence into adulthood. The global prevalence of ADHD is 3–7% [1], and approximately 5% of children [2] and 4% of adults show ADHD [3]. The main clinical features of ADHD are hyperactivity, having difficulty sustaining attention, inhibiting a prepotent response, and difficulty in holding goals and plans [4]. Due to its pervasiveness, ADHD can interfere negatively with general well-being, social life, academic performance, and development of social skills [5].

Many studies have highlighted deficits in executive functions as one of the main characteristics of ADHD, especially with regard to response inhibition, attention, and working memory [4,6,7,8,9,10,11,12,13,14,15]. Children with ADHD continue to show significant symptoms of the disorder into adulthood and are at greater risk for long-term negative outcomes, such as lower education and employment, substance abuse, and adult psychiatric disorders, than their non-ADHD peers [16].

Given the serious academic, social, familial, and accidental injury-related effects of ADHD, the need to develop and disseminate effective treatments is pressing [17].

In Europe, the guidelines that were produced by The National Institute for Health and Clinical Excellence [18,19] recommend group-based parent training/education programs or other group-based psychological treatments (e.g., cognitive-behavioral therapy, social skills training and drugs for school-aged children and young people with severe ADHD. US guidelines recommend the use of psychostimulants in all cases of moderate or severe ADHD [20,21]. However, although children with ADHD respond to medication in the short term, its long-term effectiveness is unknown [17] and the development of sustained, generalized, evidence-based interventions for ADHD is the major challenge to date.

With its focus on attention, mindfulness meditation is a promising form of training that is gaining empirical support as a complementary or alternative intervention for ADHD [22,23]. It is based on Buddhist traditions and Western knowledge of psychology, in which awareness and nonjudgmental observations of present-moment experiences are increased while automatic responding is reduced [24,25]. A fundamental action mechanism of mindfulness meditation is attention regulation [26,27,28], derived from training to sustain the focus of attention on present-moment experiences (thoughts, emotions, body sensations), attempting to gently shift the attention of participants back to themselves when they become aware that their minds have drifted from the meditation object. Mindfulness meditation has emerged as a new approach for reducing stress and an important innovative training modality in treating psychiatric and neurodevelopmental disorders.

Researching the efficacy of mindfulness training in children and adolescents is a relatively new domain (see the meta-analyses and systematic reviews [29,30,31]). Existing evidence has demonstrated that it has positive effects on psychological well-being [32,33,34,35], pain management [36], depressive symptoms and anxiety [32,37,38,39,40], negative behaviors [31] and cognitive/executive functions and attention [34,40,41,42] in children and adolescents.

Several studies have also determined the effects of mindfulness meditation training in ADHD [22,23,29]. A meta-analysis by Cairncross and Miller [22] found that mindfulness interventions significantly reduce inattention and hyperactivity/impulsivity in individuals with ADHD, irrespective of informant (self-rating and observer rating) and age. However, the effect size for the decrease in inattention was larger for adults than for children/adolescents with ADHD. The authors [22] interpreted these results with caution, based on the significant heterogeneity across studies due to the informant type (self-informant or other-informant reports) and the age of the participants.

Compared with the meta-analysis by Cairncross and Miller [22], a review by Evans et al. [29] examined the use of meditation-based interventions in a more homogenous population (participants were under 18 years old), but selected heterogeneous meditation-based interventions, and considered various outcomes. The strongest effect sizes were found for yoga and meditation and when parents and children were targeted in the intervention. Mixed evidence for self-esteem, social functioning, internalizing/externalizing symptoms, and academic performance was observed. The authors addressed several limitations in the studies that were included in the review, such as the absence of control groups, the lack of randomization of participants, the small sample sizes, limited information on the participants and selection criteria, and non-validated measures of intervention.

A more recent meta-analysis and review by Zhang et al. [23] assessed the efficacy of meditation-based interventions (mindfulness and yoga techniques) with regard to the core symptoms of ADHD and the neuropsychological deficits that are associated with it. When symptoms of ADHD were considered, meditation-based interventions were significantly more effective than the control conditions in children and adolescents. The significant effects on core symptoms of ADHD were interpreted as being the direct consequence of meditation-based interventions that typically increase attention process, self-control, and emotional regulation. In contrast, no significant effect was found for neuropsychological measures of inhibition or inattention, with evidence of heterogeneity for both measures. Similarly to earlier studies [22,29], Zhang et al. [23] raised concerns over several methodologically and clinically relevant issues in the studies that were examined, such as blinding concerns, selection bias, the lack of protocol, inconsistency between control conditions, the heterogeneous nature of the neuropsychological measures that were considered, and the simultaneous use of medication in certain participants.

In summary, preliminary studies on mindfulness training in children and adolescents with ADHD have demonstrated a positive effect on ADHD symptoms. Data derived by combining objective neuropsychological measures with parent- and self-report questionnaires is scarce, and the results that exist are controversial.

Moreover, evidence is limited by the lack of an active control group or the heterogeneity of control conditions, which varied between studies, encompassing self-guided handouts on skills, nonviolent resistance training for parents, psychoeducation, and wait list. Selection bias, lack of protocol, and the simultaneous use of medication should also urge caution in the interpretation of the results.

In considering mindfulness training in children with ADHD, studies must comprise homogeneous groups (e.g., for age, diagnosis, and pharmacological therapy) and replicable procedures (e.g., the implementation of protocols, neuropsychological measures other than self-reports and parent reports).

The present study attempted to control for issues affecting previous results on mindfulness training such as by providing an active control condition designed to be comparable with and structurally equivalent to the mindfulness-oriented meditation (MOM) program, by the combination of objective neuropsychological measures with parent and self-report questionnaires, the selection of children with ADHD with a narrow age range and no concurrent treatment, and the random assignment of participants to the MOM group (MOM G) or to the active control group.

With this aim, 32 children with ADHD aged 7–11 years were randomly assigned to the MOM G, which underwent mindfulness training or to the active control group, the Emotion Education Program group (EEP G), which entered the emotion awareness and recognition program. Participants were assessed at baseline (T0) and post-training (T1) for neuropsychological measures that involved executive functions (i.e., working memory, inhibitory control, switching, and sustained performance), ADHD symptoms, behavioral and emotional aspects, mindfulness measures, depressive and anxious symptoms, parenting stress (by using self-ratings and parent ratings), and academic skills (i.e., mental calculation and reading). None of the children had received or was receiving any pharmacological, psychological, behavioral, or educational treatment.

The MOM program, as one form of mindfulness [43], was recently proposed by Fabbro and Muratori [44], in turn inspired by the Theravada schools of Buddhism [45] and western-based mindfulness programs, such as mindfulness-based stress reduction [25,46,47,48]. The current MOM approach has been used with children [49], consisting of 3 sessions per week for 8 weeks and, consistent with previous mindfulness-meditation programs for children [34], the duration of the sessions increased gradually over time.

The EEP was designed to be comparable with and structurally equivalent to the MOM program. It was organized into 3 sessions per week for 8 weeks and the duration of the sessions followed the same progression as the MOM training. The activities of the EEP G consisted of listening to and commenting on chapters of a book to discover and be aware of various emotions.

Because mindfulness meditation programs focus on attention regulation and on reducing automatic responding, we predicted that MOM would have positive effects on neuropsychological measures that involve executive functions. We also expected to observe better effects on mindfulness measures and symptoms of ADHD, as rated by parents, in the MOM G versus EEP G. Due to the short duration of the intervention, we predicted limited effectiveness on academic skills, perceived family stress, anxiety, and depressive symptoms.

## 2. Materials and Methods

### 2.1. Participants

The estimated sample size [50] for this preliminary study was 32 participants.

Thirty-five children with ADHD (age range: 7–11; M = 8.9, *SD* = 1.2; 9 females) were recruited from a waiting list for a multimodal intervention that was based on psychological, behavioral, and educational interventions at the Child and Adolescent Neuropsychiatry Unit of the Bambino Gesù Children’s Hospital (Rome, Italy).

All participants underwent a child psychiatric and neuropsychological examination conducted by experienced developmental psychiatrists and neuropsychologists. The diagnosis of ADHD was made according to the Diagnostic and Statistical Manual of Mental Disorders (DSM)-5 criteria [2] and was based on developmental history, an extensive clinical examination, and a semi-structured interview, Schedule for Affective Disorders and Schizophrenia for School-Age Children—Present and Lifetime Version, K-SADS-PL [51], which diagnoses current and past episodes of psychopathology in children and adolescents according to DSM-IV criteria [52].

According to DSM-5 [2], the children with ADHD were characterized as follows: 30 fulfilled the diagnostic criteria for ADHD Combined presentation, 2 had ADHD predominantly Hyperactive-Impulsive presentation, and 3 had ADHD predominantly Inattentive presentation.

Global functioning was assessed with the Children’s Global Assessment Scale (C-GAS) [53]. The C-GAS estimates the overall severity of disturbance (range, 0–100). Scores over 90 indicate superior functioning, whereas scores under 70 suggest impaired global functioning.

IQ was measured by using the Wechsler Intelligence Scale for Children-IV (WISC-IV, Italian edition) [54] or Colored Progressive Matrices [55].

Children with ADHD who were included in the study met the following criteria: (a) a primary diagnosis of ADHD, according to the criteria of DSM-5 [2]; (b) age between 7 and 11 years; (c) IQ ≥ 85.

The exclusion criteria were as follows: (a) the presence of neurological and neurosensory deficits; (b) the presence of comorbid psychopathological disorders or autism spectrum disorder; (c) past or present drug treatment, cognitive behavioral therapy, training/education program, or any other group-based psychological treatment for parents.

All participants and their parents gave written informed consent after receiving a comprehensive description of the study. This study was performed in accordance with the Declaration of Helsinki and was approved by the local ethical committee of the Bambino Gesù Children’s Hospital (Process Number 1162/2016).

### 2.2. Procedure

**Figure 1:**
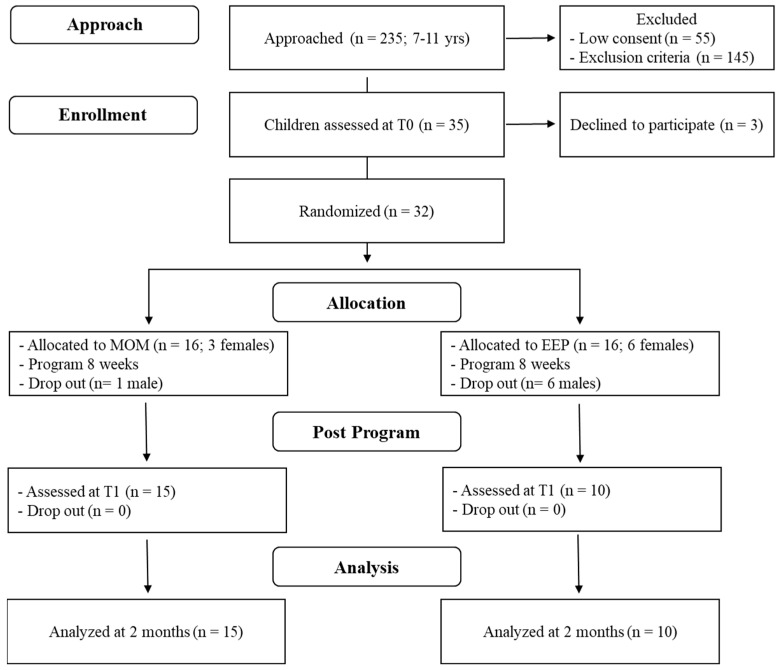
Study flow diagram.

This randomized study comprised two arms (see Figure 1). The baseline assessment (T0) was conducted twice at the Child and Adolescent Neuropsychiatry Unit of the Bambino Gesù Children’s Hospital by two child developmental psychologists who were blind to the interventions, with each evaluation lasting approximately 1.5 h. After completing the baseline assessment, three children declined to participate, and ultimately, 32 participants (23 males) were allocated to the two arms (MOM program or EEP), based on simple random allocation using a computer-generated random number sequence that was performed by clinical staff members who were not involved in the research. The MOM G was composed of 16 children (3 females) who underwent mindfulness meditation training, and the EEP G comprised 16 children (6 females) who participated in an EEP on listening to and commenting on a book on the importance of feeling positive and negative emotions.

Both of the training programs were provided at the Child and Adolescent Neuropsychiatry Unit of the Bambino Gesù Children’s Hospital. Before the training began, one participant in the MOM-G and six in the EEP G dropped out.

At T0, the two groups did not differ in chronological age (CA MOM G: M = 8.9, *SD* = 1.3; CA EEP G: M = 9, *SD* = 1.2), IQ (IQ MOM G: M = 109.9, *SD* = 11.1; IQ EEP G: M = 104.4, *SD* = 8.2), or C-GAS score (C-GAS MOM G: M = 53.4, *SD* = 2.6; C-GAS EEP G: M = 53.5, *SD* = 1.8).

The post-training evaluations (T1) were conducted twice within two weeks after the end of the training by two child developmental psychologists who were blind to the interventions, with each session lasting approximately 1.5 h.

### 2.3. Mindfulness-Oriented Meditation Training

The MOM program was conducted by two qualified mindfulness meditation instructors (VC and OS). A developmental psychologist (VZ) was also present to help children with ADHD to follow the sessions.

The training was inspired by previous MOM interventions for clinical and nonclinical adult and child populations [44,49,56,57,58], which were in turn based on the Mindfulness Based Stress Reduction protocol [25,47]. The MOM protocol consisted of three sessions per week for eight weeks [49]. Consistent with earlier mindfulness meditation programs for healthy children [34], the duration of the sessions increased gradually over the eight weeks, starting from 6 min and rising to 30 min. For the first two weeks, the MOM training lasted for less than 10 min at each meeting, rising to 30 min at the end of the course (Week 8). The reason for this adaptation was the lower attentional capacity of children with ADHD and their difficulty in engaging in a single activity for long periods.

Each session was divided into a series of three meditation exercises that focused on three types of activity: (i) mindfulness of breathing, (ii) mindfulness of body parts, and (iii) mindfulness of thoughts and emotions. After the meditation exercise, there was a brief debriefing phase that lasted approximately half of the time that was dedicated to the meditation exercises to allow the children to express their feelings and pose questions about the exercise that had just been completed.

Specifically, the three meditation activities were proposed to the MOM G as “games” that were meant to promote awareness of breath, body parts, and thoughts. In each of the three weekly sessions, children with ADHD were first required to concentrate on breath. In the second meditation exercise, participants had to focus their attention on various body parts. In the last activity, children were encouraged to observe the stream of their thoughts and emotions. For a brief description of the structure of the sessions and activities included in the MOM training, see Supplementary Materials. At the end of each session, children were recommended to practice meditation wherever they were (several times per day) in order to generalize the gains that were made in training to daily life. During the eight weeks of training, children were given homework (“Meditation Diaries”) and instructed to write about their meditation experiences in everyday life.

### 2.4. Emotion Education Program

The activities of the EEP G were designed to be comparable with and structurally equivalent to those of the MOM program (see MacCoon et al. [59] and Crescentini et al. [49]). Similar to the MOM training, the EEP was organized into three meetings per week for eight weeks.

The duration of the sessions followed the same progression as in the MOM training: for the first two weeks, the EEP lasted for less than 10 min at each session, rising to 30 min at the end of the course. The activities of the EEP G consisted of listening to and commenting on chapters of the book “Six Pixies in My Heart” (“Sei Folletti nel Mio Cuore”) [60].

The program was led by the same trainers as in the MOM training. The book is about a shy and sensitive child who decides to avoid all of his emotions to avoid being defined as “sensitive” by his friends and schoolmates. However, at the end of the book, the child learns the importance of feeling positive and negative emotions in his heart and appreciates that he is sensitive.

During the sessions, the trainers asked the children to discuss the stories, the emotions that they felt, and the physical sensations that were associated with these emotions. The activities of listening to and commenting on the chapters allowed children to discover various emotions that can be experienced in many situations and to consider and be aware of their own emotions. At the end of each session, the children were recommended to pay attention to the emotions that were experienced in their everyday life in order to generalize the gains that were made in training to daily life.

During the eight weeks of the EEP, participants were given homework (“Diaries”) and instructed to write down the situations in which they were aware of their emotions. For a brief description of the structure of the sessions and activities included in the EEP, see Supplementary Materials.

In summary, EEP was an active control condition for the MOM program because it shared several crucial elements with MOM, including timing and setting, requests for silence, group work, interaction between children and trainers, and the assignment of homework, but was designed not to be related to the practice of mindfulness [59].

## 3. Measures

Children from both groups were assessed at T0 and T1 with regard to the following.

### 3.1. Neuropsychological Measures

The Continuous Performance Test-II (CPT-II) 5th version [61] is a computerized measure of sustained performance. Children were required to press the spacebar when any letter except “X” appeared on the screen. Measures of CPT-II mean correct Hit Reaction Times (CPT-II HRT) and the standard deviation of correct CPT-II HRTs (CPT-II HRT-SD) were included in the analyses.

In the Stroop Color Word Test [62], children were instructed to read the words (cond1), name the colors (cond2), and, finally, name the color of the ink of the printed words when they were incongruous (cond3) as quickly and as accurately as possible. To calculate the cost of an incongruous response, the time for cond2 was subtracted from that of cond3 (cond3-cond2), to calculate the number of errors (cond3-cond2). To integrate time and the proportion of errors, an Inverse Efficiency Score (STROOP IES) was calculated [63] as follows: time (cond3-cond2)/(1-proportion of errors cond3-cond2), where the proportion of errors was calculated, based on the number of stimuli (*n* = 100).

The stop task [64,65,66,67] consists of randomly intermixed Go and Stop Trials. In Go trials, children were instructed to press the spacebar as quickly as possible after the appearance of the go signal. In stop trials, after a variable delay (stop signal delay, SSD), a stop signal (red way stop) appeared after the Go signal, and children were instructed to refrain from responding. The Stop Signal Reaction Time (SSRT) was estimated (in msec) by subtracting the mean estimate of SSDs from the observed mean of the reaction times in no-stop trials.

In the N-Back task, children were presented with a single blue square at any location in a 3 × 3 grid on a computer screen and instructed to make a decision (yes/no) by pressing a button with regard to whether the current stimulus was in the same location as that presented N-positions earlier. Only when the accuracy was 80% was the next N-back level submitted. A N-Back Inefficiency Index (N-BACK II) was calculated, based on the percentage of errors in the last not-achieved n-back.

### 3.2. Parent and Self-Report Questionnaires

Conners’ Parent Rating Scales Long Version Revised (CPRS-R:L) [68] is composed of behavior rating scales that are commonly used to assess behaviors that are related to ADHD and other disorders in children. It is completed by parents to obtain a measure of hyperactivity and inattention symptoms for ADHD. The CPRS-R:L comprises 7 subscales that have been derived by factor analysis: oppositional, cognitive problems/inattention, hyperactivity, anxious-shy, perfectionism, social problems, and psychosomatic problems. In addition, ADHD index, Conners’ Global Index (CGI) Restless-Impulsive, CGI Emotional Lability, CGI Total, DSM-IV Inattentive, DSM-IV Hyperactive/Impulsive, and DSM-IV Total scores were calculated. The cutoff for T-scores for clinical significance was >70 (very elevated). T-scores from 60–70 were considered to be high average or elevated.

The Child Behavior Checklist for Ages 6–18 (CBCL 6-18) [69] is a paper-and-pencil-based questionnaire of child and adolescent behaviors and emotions that is completed by parents and comprises 8 syndrome scales (anxious/depressed, withdrawn/depressed, somatic complaints, social problems, thought problems, attention problems, rule-breaking behavior, and aggressive behavior), 3 broadband scores (internalizing and externalizing problems, and total problem), DSM-oriented scales (affective problems, anxiety problems, somatic problems, attention deficit/hyperactivity problem, oppositional defiant problems, conduct problems), and the 2007 Scales (sluggish cognitive tempo, obsessive-compulsive problems, post-traumatic stress problems). The CBCL 6-18 generates a T-score for each subscale. According to normative data, a T-score above 64 was considered to be significant for the 3 broadband scales, whereas for the syndrome scales, the cut-off for clinical significance was 70.

The Multidimensional Anxiety Scale for Children (MASC) [70] is a 39-item, paper-and-pencil-based self-reported measure that performs a multidimensional assessment of anxiety in children and adolescents. Total raw scores were converted to T-scores.

The Children’s Depression Inventory (CDI) [71] is a 27-item self-reported inventory that measures depressive symptoms in children and adolescents. A score above 19 suggests the presence of clinically significant depressive symptoms.

The Child and Adolescent Mindfulness Measure (CAMM) [72] is an awareness scale for persons aged 6–18 years that detects their level of awareness. Higher total scores reflected a greater level of acceptance and mindfulness.

The Parenting Stress Index-Short Form (PSI-SF) [73,74,75] is a 36-item questionnaire that measures various aspects of perceived stress in the parenting role. A total stress score (PSI TOT) represents an index of the parent’s overall perception of parenting stress. Raw scores were converted to percentiles.

### 3.3. Academic Skills

Text Reading (MT-2) [76]. Reading speed (the mean number of syllables per second) and accuracy (number of errors) were calculated. For both reading measures, scores were split into two categories (medium/high or low/poor) according to normative data.

Mental Calculation (Batteria per la Discalculia Evolutiva (BDE)) [77,78]. The ability to compute arithmetic facts was evaluated (sum and subtraction with numbers up to ten). Children were required to solve calculations that were posed by the clinician within 2 s. The number of errors was calculated and transformed into Z-scores, based on normative data on education level.

## 4. Results

Concerning Neuropsychological Measures (see Table 1), a repeated measures ANOVA was conducted on the five neuropsychological measures as the dependent variables (CPT-II HRT, CPT-II HRT-SD, STROOP IES, SSRT, and N-BACK II) with Group (MOM G, EEP G) and Time (T0, T1) as the independent variables. Significant effects for Group (F_1,23_ = 4.8, *p* = 0.039, ηp2 = 0.17), Time (F_1,23_ = 8.1, *p* = 0.009, ηp2 = 0.26), Task (F_4,92_ = 234.4, *p* < 0.0001, ηp2 = 0.91), and Group × Time (F_1,23_ = 5.98, *p* = 0.023, ηp2 = 0.21) were found. However, Group × Task (F_4,92_ = 1.38, *p* = 0.25, ηp2 = 0.06), Task × Time (F_4,92_ = 1.53, *p* = 0.2, ηp2 = 0.06), and Group × Task × Time interactions were not significant (F_4,92_ = 1.3, *p* = 0.27, ηp2 = 0.05).

The post hoc analysis (Unequal N Tukey’s Honestly Significant Difference test) of the effect of Group × Time showed that MOM G mean scores decreased from T0 to T1 (*p* = 0.0028), while EEP G mean scores did not change from T0 to T1 (*p* = 0.99). Moreover, the two groups differed at T0 (*p* = 0.027) but not at T1 (*p* = 0.99).

Concerning ADHD symptoms (see Table 2), a repeated measures ANOVA was conducted on CPRS-R:L, with T-scores of 14 subscales as the dependent variables, and Group (MOM G, EEP G) and Time (T0, T1) as the independent variables. Significant effects for Group (F_1,23_ = 6.49, *p* = 0.018, ηp^2^ = 0.22), Subscale (F_13,299_ = 42.8, *p* < 0.0001, ηp^2^ = 0.65), Group × Subscale (F_13,299_ = 1.92, *p* = 0.028, ηp^2^ = 0.08), and Group × Time × Subscale interactions (F_13,299_ = 1.8, *p* = 0.04, ηp^2^ = 0.07) were found. The main effect of Time (F_1,23_ = 2.28, *p* = 0.14, ηp^2^ = 0.07) and Group × Time interaction (F_1,23_ = 2.17, *p* = 0.15, ηp^2^ = 0.15) were not significant. As shown by the post hoc analysis of Group × Time × Subscale interaction (Unequal N Tukey’s Honestly Significant Difference test), only scores in the MOM G decreased from T0 to T1 for the CGI Restless-Impulsive and CGI Total subscales (*p* = 0.04 and *p* = 0.023, respectively).

Further, the change between T0 and T1 for the CGI Restless-Impulsive and CGI Total subscales had clinical significance only in the MOM G: the subscales scores decreased, on average, from a clinical level at T0 (mean T-scores higher than 70) to a borderline range at T1 (between 60 and 70). Although no effect of the training program was observed for the Oppositional subscale, the change between T0 and T1 had clinical significance only in the MOM G: Oppositional subscale scores decreased, on average, from a borderline range at T0 (mean T-scores between 60 and 70) to a typical level at T1 (lower than 60). Similarly, although training did not have any effect on it, DSM-IV Hyperactive/Impulsive subscale scores between T0 and T1 declined, on average, only in the MOM G, from a clinical level at T0 (mean T-scores above 70) to a borderline range at T1 (between 60 and 70).

For the CBCL 6-18, a repeated measures ANOVA was conducted on T-scores of the 24 subscales as the dependent variables, and Group (MOM G, EEP G) and Time (T0, T1) as the independent variables. Neither the Group (F_1,23_ = 3.14, *p* = 0.09, ηp^2^ = 0.12), Time (F_1,152_ = 1.181, *p* = 0.29, ηp^2^ = 0.05), Group × Time (F_1,23_ = 1.72, *p* = 0.20, ηp^2^ = 0.07) nor the Group × Time × Subscale interactions (F_23,529_ = 1.00, *p* = 0.46, ηp^2^ = 0.04) were significant. However, the main effect of Subscale (F_23,529_ = 74.48, *p* < 0.0001, ηp^2^ = 0.76) and the Group × Subscale interaction were significant (F_23,529_ = 1.79, *p* = 0.01, ηp^2^ = 0.07). 

To determine the effects of MOM on parent and self-report questionnaires on anxiety, depressive symptoms, mindfulness, and parenting stress, a repeated measure ANOVA was performed, with MASC, CDI, CAMM, and PSI-SF scores as the dependent variables, and Group (MOM G, EEP G) and Time (T0, T1) as the independent variables. The main effects of Group (F_1,23_ = 0.21, *p* = 0.65, ηp^2^ = 0.01) and Time (F_1,23_ = 0.04, *p* = 0.84, ηp^2^ = 0.01) were not significant. The Group × Time (F_1,23_ = 0.60, *p* = 0.44, ηp^2^ = 0.02) and Group × Time × Task interactions (F_3,69_ = 0.16, *p* = 0.92, ηp^2^ = 0.01) were also not significant. However, the main effect of Task (F_3,69_ = 96.15, *p* < 0.0001, ηp^2^ = 0.8) was significant.

Concerning academic skills, a repeated measure ANOVA was performed on mental calculation Z-score as the dependent variable, and Group (MOM G, EEP G) and Time (T0, T1) as the independent variables. The main effect of Group (F_1,23_ = 5.37, *p* = 0.03, ηp^2^ = 0.2) was significant but Time effect (F_1,23_ = 3.6, *p* = 0.07, ηp^2^ = 0.1) and Group × Time interaction (F_1,23_ = 0.23, *p* = 0.63, ηp^2^ = 0.01) were not significant.

Chi-squared test was used to compare the number of participants in the two groups whose reading performance changed (medium/high or low/poor) from T0 to T1 with regard to reading speed or reading accuracy. For text reading also, the number of participants in the two groups whose reading performance changed from T0 to T1 with regard to speed (χ^2^_3_ = 0.27, *p* = 0.96) or accuracy (χ^2^_3_ = 3.49, *p* = 0.32) did not differ.

### Discussion

The aim of this study was to evaluate the effects of an eight-week MOM program on neuropsychological measures, ADHD symptoms, behavioral and emotional aspects, depressive and anxious symptoms, a mindfulness measure, a parenting stress index, and academic skills in children with ADHD.

On average, MOM training had positive effects, with a large effect size, on neuropsychological measures (Group × Time effect), as evidenced by the average responses on neuropsychological measures improving significantly at T1 compared with T0 in the MOM G but not in the EEP G.

With regard to ADHD symptoms, only the MOM G had significantly lower T-scores at T1 than at T0 on the CGI Restless-Impulsive and CGI Total subscale scores (Group × Time × Subscale), with a small effect size. Moreover, the changes in ADHD symptoms after MOM training had clinical significance, based on parent rate T-scores declining on several CPRS-R:L subscales.

The significant mean improvement in all neuropsychological measures after training in the MOM G suggested that MOM training could appreciably reduce executive functions deficits in ADHD. Our tasks involved higher-order cognitive abilities, such as working memory, inhibitory control, switching, and sustained performance. However, there are few data on the association between mindfulness and executive functions [79,80,81] in typically developing children [42] and in children with neurodevelopmental disorders, having been studied primarily in adult populations and adolescents [82,83].

Concerning studies on typical populations, our results are in line with findings that showed an association between inhibition, working memory and mindfulness. Specifically, Riggs et al. [83] tested a model considering the association of different executive functions and mindfulness, ascertaining that only inhibition and working memory had unique associations with mindfulness in early adolescence.

The other two studies investigating working memory in relation to mindfulness in healthy adolescents [83,84] documented positive effects of mindfulness meditation interventions on working memory. It has been suggested [84] that individuals with greater working memory efficiency might be more able to remember their intention to maintain present moment awareness and, thus, be more mindful.

However, the review by Gallant [85] on the association between executive functions and mindfulness trainings showed that the effect of mindfulness interventions was more consistent for inhibition than other executive functions. It has been suggested that inhibition, in mindful awareness, does not consist of simply suppressing unwanted thoughts and behaviors, but, rather, of letting go of distractions and immersing oneself in the present moment [79].

A recent study [42] evaluated the relationship between mindfulness and executive functions in typically developing children by correlating child ratings on mindfulness and parent ratings on children’s executive functions. Although the study was limited in that it used survey data, it reported a negative correlation between mindfulness and difficulties with inhibition, working memory, and shifting, confirming that children who were more mindful were less likely to experience such difficulties. Our results strengthened those correlational findings obtained indirectly by using child and parent ratings [42]. Indeed, by administering mindfulness training and analyzing its effects on neuropsychological measures that involve executive functions, we directly verified the relationship between mindfulness and executive functions.

To date, it is difficult to compare our results with other findings on ADHD because only few studies have focused on the association between mindfulness and executive functions in children/adolescents with ADHD. No significant effects of mindfulness interventions were found in a review [23] on neuropsychological measures of inhibition and working memory in children and adolescents with ADHD but we would urge caution in the interpretation of these results since the review was based on three studies only.

With regard to ADHD symptoms, our results were in line with a recent meta-analysis aimed at investigating the efficacy of mindfulness-based interventions on ADHD core symptoms in comparison with active control conditions [86]. The meta-analysis suggested that mindfulness-based interventions had large effects in reducing ADHD core symptoms. Authors interpreted these results as indicating that mindfulness-based exercise, which emphasized more the nonjudgmental attention of participants to occurring experience in the present moment, improves attentional regulation.

Looking at the effect sizes, our results also indicated that the effects of MOM training on neuropsychological measures were stronger than on parent ratings.

In general, the positive outcomes of MOM training on neuropsychological measures and ADHD symptoms that we found could be explained by considering that our program focused on three meditation exercises that allocated attentional resources to breath, various body parts, and thoughts. Thus, during MOM, our children with ADHD developed self-regulatory skills, especially attentional control, to maintain focus on present moment experiences and inhibit distractions [79].

Our results, although they are derived from a small group of children with ADHD, are strengthened by the inclusion of an active control group (the EEP group) that helps to control the alternative interpretation of the possible effects of the MOM intervention. As previously suggested ([86]), the inclusion of an active control group improves the internal validity of findings. Inactive conditions (i.e., treatment as usual and wait-list) have a significant impact on the heterogeneity of the results of mindfulness-based interventions on ADHD, and contributed to a smaller effect on ADHD core symptoms, when compared to active conditions.

Our study has some limitations. As a preliminary study, the number of participants was limited, but the results are encouraging and helpful in designing and executing a large-scale clinical trial on MOM. Recruiting more participants could remediate the possible bias of gender in our study, and children could be more equally distributed between the two conditions with regard to demographics such as gender.

Another limitation was the lack of multiple informants. Parents would/should be aware of the intervention condition to which their child was assigned, which would likely affect their ratings. Including several informants, such as teachers, would have helped with this problem.

Our study lacked also of a follow-up period, which should be included in future studies to verify the duration of changes in the MOM G.

Future studies should also understand why other measures, such as some behavioral and emotional aspects, depressive and anxiety symptoms, the mindfulness measure, parenting stress index, and academic skills, did not change after mindfulness practice, and should determine whether a longer duration of training would thus be more useful.

Our study is a preliminary step towards establishing the effectiveness of MOM intervention in ADHD and future studies are needed to confirm the benefits of MOM by including more participants, using longer training periods, combining neuropsychological measures with questionnaires from multiple respondents (child, parent, and teacher), and comparing results from groups of parents who do not participate in MOM interventions (as in our study) versus those who are asked to meditate with their children [87] or undergo mindful parent training in parallel [88].

## 5. Conclusions

When comparing our study with previous works on mindfulness training in children and adolescents with ADHD, it is one of the few that combined objective neuropsychological measures with parent- and self-report questionnaires. Moreover, our study included an active control condition (focused on emotion awareness and recognition) that was structurally equivalent to the mindfulness-meditation program, and we randomly assigned to the two conditions children with ADHD with a narrow age range and no concurrent treatment.

Overall, our results are encouraging and suggest that mindfulness meditation practices that are performed for a short period (eight weeks) promote changes in neuropsychological measures, especially those in which executive functions are involved, and behavioral symptoms in children with ADHD.

## Figures and Tables

**Table 1:** Means and Standard Deviations of Neuropsychological Measures in the Mindfulness-Oriented Meditation Group and in the Emotion Education Program Group.

		MOM G	EEP G
Neuropsychological Measure	Time	M (*SD*)	M (*SD*)
CPT-II HRT (msec)	T0	505.39 (73.00)	445.37 (75.98)
	T1	431.33 (52.26)	428.21 (45.08)
CPT-II HRT-SD (msec)	T0	348.23 (89.39)	238.76 (94.94)
	T1	222.12 (93.04)	239.97 (65.26)
STROOP IES (msec/errors)	T0	115.48 (47.86)	110.76 (57.90)
	T1	96.62 (41.91)	107.26 (35.12)
SSRT (msec)	T0	347.24 (190.72)	279.62 (106.35)
	T1	319.74 (62.14)	271.39 (62.94)
N-BACK II (% of errors)	T0	55.67 (23.65)	36.40 (16.97)
	T1	48.93 (14.31)	45.50 (15.45)

Note. MOM G = Mindfulness-Oriented Meditation Group; EEP G = Emotion Education Program Group; CPT-II = Continuous Performance Test-II; HRT = Hit Reaction Times; HRT-SD = Hit Reaction Times-Standard Deviation; STROOP IES = Stroop Color Word Test Inverse Efficiency Score; SSRT = Stop Signal Reaction Time; N-BACK II = N-Back Inefficiency Index; T0 = baseline; T1 = post-training.

**Table 2:** Means and Standard Deviations of Conners’ Parent Rating Scales Long Version Revised subscales in the Mindfulness-Oriented Meditation Group and in the Emotion Education Program Group.

		MOM G	EEP G
CPRS-R:L Subscale	Time	T-Score M (*SD*)	T-Score M (*SD*)
Oppositional	T0	64.13 (14.58)	67.90 (12.64)
	T1	56.47 (10.64) ^b^	68.90 (14.16)
Cognitive Problems/Inattention	T0	76.80 (12.45)	86.20 (11.00)
	T1	75.00 (11.85)	84.00 (14.37)
Hyperactivity	T0	69.80 (14.51)	81.00 (12.02)
	T1	62.13 (10.98)	79.00 (13.22)
Anxious-Shy	T0	53.40 (13.89)	54.20 (13.07)
	T1	49.73 (11.50)	56.80 (12.85)
Perfectionism	T0	51.93 (9.25)	50.10 (8.37)
	T1	43.47 (7.08)	50.80 (7.54)
Social Problems	T0	63.40 (16.05)	64.80 (19.36)
	T1	65.80 (14.63)	61.50 (15.09)
Psychosomatic Problems	T0	55.93 (19.64)	60.80 (16.10)
	T1	48.93 (13.62)	64.90 (16.94)
ADHD Index	T0	78.80 (12.70)	83.80 (6.73)
	T1	71.47 (10.13)	80.50 (14.42)
CGI Restless-Impulsive	T0	73.47 (14.29)	76.90 (9.42)
	T1	63.93 (10.12) ^a,b^	79.60 (11.17)
CGI Emotional Lability	T0	56.20 (14.66)	67.10 (12.74)
	T1	48.73 (8.94)	67.80 (17.67)
CGI Total	T0	70.40 (14.83)	76.70 (9.92)
	T1	60.53 (9.88) ^a,b^	78.80 (13.20)
DSM-IV Inattentive	T0	78.66 (12.02)	84.60 (10.69)
	T1	74.80 (12.04)	84.20 (14.57)
DSM-IV Hyperactive/Impulsive	T0	70.00 (12.45)	77.10 (9.47)
	T1	63.13 (10.49) ^b^	75.20 (14.77)
DSM-IV Total	T0	77.07 (12.65)	84.30 (9.94)
	T1	71.47 (10.53)	82.50 (14.76)

Note. CPRS-R:L = Conners’ Parent Rating Scales Long Version Revised; MOM G = Mindfulness-Oriented Meditation Group; EEP G = Emotion Education Program Group; CGI = Children Global Index; T0 = baseline; T1 = post-training. ^a^ Statistical difference between T0 and T1 (*p* ≤ 0.05). ^b^ Clinical change between T0 and T1.

## Supplementary Materials

The following are available online at https://www.mdpi.com/1660-4601/17/18/6916/s1, Table S1: Overview of the activities included in the Mindfulness-Oriented Meditation training and in the Emotion Education Program.
