# Supplementary material for: Clinical Application of Mindfulness-Oriented Meditation: A Preliminary Study in Children with ADHD
Source: Int J Environ Res Public Health. 2020 Sep 22;17(18):6916. doi: 10.3390/ijerph17186916 (PMC7557753; doi:10.3390/ijerph17186916)
Supplement: Supplementary file 1 [file ijerph-17-06916-s001.pdf]

Table S1: Overview of the activities included in the Mindfulness-Oriented Meditation Training and Emotion Education Program.

Week	Timing per day (min)	Timing per week (min)	Mindfulness-Oriented Meditation (MOM)	Emotion Education Program (EEP)
**1**	6	18	• Mindfulness of breathing: feeling the breath. Place the hand on the abdomen and observe the abdomen moving while breathing.<br>• Mindfulness of body parts: listening to the sounds of the body and to the sounds of the room.<br>• Mindfulness of mental states: observing thoughts, as they were soap bubbles.	Chapter 1-2<br>Following the protagonist's story, try to listen to the heart and try to feel the emotions laying inside.<br>Listening to the heart and trying to associate the feelings with the pixies described in the book.<br>Writing the emotions of the moment.
**2**	9	27	• Mindfulness of breathing: feeling the breath. Place the hand on the abdomen and observe the abdomen moving while breathing.<br>• Mindfulness of body parts: slowly walking, feeling and observing all foot support points.<br>• Mindfulness of mental states: observing thoughts, as they were clouds in the sky. Trying to see the main thought - as a cloud - in the mind.	Chapter 3-4-5<br>Listening to the heart and trying to associate the feelings with the pixies described in the book.<br>Writing the emotions of the moment<br>Writing the thoughts and the emotions after listening to the book.

**3**	12	36	• Mindfulness of breathing: feeling a mate's breath putting the hands on his/her abdomen.<br>• Mindfulness of body parts: taking a mate's hand and feeling the contact.<br>• Mindfulness of mental states: observing thoughts, as they were sea waves. Trying to see the main thought - as waves - in the mind.	Chapter 6-7<br>Listening to the heart and trying to associate the feelings with the pixies described in the book.<br>Writing the emotions at the moment, and the relative physical sensations.<br>Writing the thoughts and the emotions after listening to the book.<br>Try to draw the physical sensations.
**4**	15	45	• Mindfulness of breathing: trying to feel the breath in the nose, without controlling it.<br>• Mindfulness of body parts: touching the different parts of the own face and mate's face.<br>• Mindfulness of mental states: observing thoughts, as they were soap bubbles, clouds in the sky, sea waves. Trying to see the path of a thought: where it comes from, how it disappears and what emotions it arouses.	Chapter 8-9<br>Listening to the heart and trying to associate the feelings with the pixies described in the book.<br>Writing the emotions at the moment, and the relative physical sensations.<br>Try to draw where emotions come from.
**5**	18	54	• Mindfulness of breathing: trying to think the word "in" when air enters the nose, "out" when air comes out from the nose.<br>• Mindfulness of body parts: imaging an object and drawing it. Then, observing the chosen object in details and drawing it again.<br>• Mindfulness of mental states: thinking to himself, a mate or a relative and mentally addressing some friendly wishes (peace, happiness, health).	Chapter 10-11-12<br>Listening to the heart and trying to associate the feelings with the pixies described in the book.<br>Writing the emotions at the moment, and the relative physical sensations.<br>Try to draw the path of the emotions: where they come from and where they go, if they disappear.

**6**	21	63	• Mindfulness of breathing: trying to feel the difference of breath by putting the hands on the nose, on throat, on the abdomen.<br>• Mindfulness of body parts: trying to smell, watch, and touch the raisin before eating it (Raisin meditation).<br>• Mindfulness of mental states: drawing a mate imagining him in a place where he feels happy, peace, loved and healthy. Looking into eyes, mentally addressing some friendly wishes (peace, happiness, health).	Chapter 13-14-15<br>Listening to the heart and trying to associate the feelings with the pixies described in the book.<br>Writing the emotions at the moment, and the relative physical sensations.<br>Try to draw the path of the emotions: where they come from and where they go, if they disappear.
**7**	24	72	• Mindfulness of breathing: imaging a little man in the nose that moves, following the air coming in and out.<br>• Mindfulness of body parts: imaging to be a seaweed, frog, and moving like it. Observing the movements. Laying down pretending to be a paper and imaging to scan the body, as if you were in a copying machine.<br>• Mindfulness of mental states: drawing a mate imagining him in a place where he feels happy, peace, loved and healthy. In pairs, representing (for example graphically) the emotion experienced during the experience and write a thought about it.	Chapter 16-17-18<br>Listening to the heart and trying to associate the feelings with the pixies described in the book.<br>Writing the emotions at the moment, and the relative physical sensations.<br>Try to find a still and quiet place in the heart to enter when feeling overwhelmed by emotions.

**8**	30	30	• Mindfulness of breathing: feeling the points that air touches when it comes in and out of the nose.<br>• Mindfulness of body parts: imaging to be a lizard exposed to the sun and observing each movement of the body. Laying down pretending to be a paper and imaging to scan the body, as if you were in a copying machine. Walking meditation. Feeling and observing all movements.<br>• Mindfulness of mental states: visualizing thoughts as a masquerade parade. Observing them and noticing that they are external to the person. Drawing them, observing the differences between thoughts. Imaging a quiet place in the heart (Still Quiet Place) to enter when feeling overwhelmed by emotions. In a circle looking at each other's eyes and imagining in silence the mates feeling (To explore perception). Trying to feel the emotions related to the main thought of the moment. Drawing or writing the emotions and thoughts.	Chapter 19-20-21<br><br>Listening to the heart and trying to associate the feelings with the pixies described in the book.<br><br>Writing the emotions at the moment, and the relative physical sensations.<br><br>Try to find a still and quiet place in the heart to enter when feeling overwhelmed by emotions.

Notes: A brief description of the activities is given for each week. Activities remained constant for the 3 meetings of a week. In the Emotion Education Program (EEP) “Chapter” refers to the book “Six pixies in my heart” (Corallo 2011). See also Crescentini et al. (2016).
